# Bronchial epithelial cells cultured from clinically stable lung allograft patients promote the development of macrophages from monocytes rather than dendritic cells

**DOI:** 10.1136/thx.2008.104067

**Published:** 2008-01-20

**Authors:** C Ward, K Eger, J Diboll, D Jones, M A Haniffa, M Brodlie, A Fisher, J L Lordan, P A Corris, C M U Hilkens

**Affiliations:** 1 Applied Immunobiology and Transplantation Research Group, Institute of Cellular Medicine, Newcastle University, Newcastle upon Tyne, UK; 2 The Musculoskeletal Research Groups, Institute of Cellular Medicine, Newcastle University, Newcastle upon Tyne, UK

## Abstract

**Background:**

It is understood that chronic allograft failure occurs as a result of alloimmune and non-alloimmune injury. Dendritic cells (DC) are thought to be crucial in regulating (allo)immune airway damage and interactions with epithelial cells are likely. Studies in human lung transplantation are limited, however, and the available literature on DC is inconsistent. This study focused on the ex vivo influence of primary bronchial epithelial cells derived from lung allografts on DC differentiation.

**Methods:**

Epithelial cell conditioned media (ECCM) were added to monocytes differentiating into DC under the influence of interleukin-4 and granulocyte macrophage-colony stimulating factor. The resultant cells were compared with DC cultured without ECCM and with monocyte-derived macrophages. Expression of typical DC (eg, CD1a) and macrophage (eg, CD14) markers was assessed by flow cytometry. Phenotypical assessments were complemented by functional studies of mannose receptor-mediated phagocytosis (FITC-dextran uptake) and antigen-presenting capability (mixed lymphocyte reactions).

**Results:**

Cells exposed to ECCM expressed significantly lower levels of CD1a than unexposed DC. CD14 expression and phagocytic function were increased. ECCM cultured cells also expressed lower levels of T cell co-stimulatory molecules, secreted an anti-inflammatory cytokine profile and had significantly reduced antigen-presenting capability.

**Conclusion:**

Using phenotypic and functional approaches, this study has shown that ECCM from lung allografts drives the production of macrophage-like cells from monocytes rather than DC. The data suggest that epithelial cells may restrain airway DC and potential alloimmunity. It is unclear whether the observed effect is specifically seen in lung transplant recipients or is a general property of bronchial epithelial cells. This may reflect a homeostatic inter-relationship between airway epithelial and DC populations relevant both to lung allografts and the lung more generally.

Lung transplantation is an accepted treatment for end stage lung disease in carefully selected patients, but success is compromised by chronic allograft dysfunction.^1^ Episodes of acute rejection are consistently identified as a key risk factor for chronic allograft dysfunction in International Registry data. This devastating complication is referred to as chronic rejection, reflecting the current understanding that chronic alloimmune damage is a key mechanism.^1^ Dendritic cells (DC) are the professional antigen-presenting cells of the airway that initiate and regulate immune responses,^2^ and DC are thought to be crucial in regulating (allo)immune damage in man. Studies in human lung transplantation are limited, however, and the available literature is inconsistent.

Yousem *et al* showed increased numbers of DC in chronically rejecting lung allografts,^3^ and a subsequent study by Leonard *et al* indicated that DC co-expressing the co-stimulatory molecules CD80 and CD86 were more frequent in allografts with chronic rejection.^4^ In contrast, a previous study from our centre showed that there were fewer CD1a-positive DC in lung allografts than in normal lungs.^5^

The airway epithelium is a recognised focus of damage in chronic allograft dysfunction, but it is also increasingly accepted that airway epithelial cells may play a key part as effector cells through the production of a wide range of inflammatory and immunomodulatory cytokines and growth factors.^6^ Physiologically, airway DC are thought to sample the airway lumen continuously for potential pathogens by projecting dendrites through the airway epithelium,^7^ and this close spatial arrangement emphasises the potential for epithelial cells to interact with and modulate DC function.^8^ Regamey *et al*^9^ have recently studied this potential using conditioned media produced from alveolar and epithelial cell lines. They showed that epithelial cells—both constitutively and following stimulation with pro-inflammatory cytokines such as tumour necrosis factor α (TNFα)—drove the production of DC from monocytes in an interleukin (IL)-15-dependent manner. IL15 is a cytokine that shares biological properties with IL2, and recombinant IL15 has been shown to cause the production of DC from monocytes. A potential implication of this study is that, in lung transplantation, airway epithelial cells may promote a situation which is deleterious to the graft, with an expanded population of DC capable of orchestrating alloimmune injury.

To our knowledge, there are no studies in primary epithelial cell cultures from lung allografts evaluating the potential for epithelial cells to modulate the production of dendritic cells from monocytes. In this study, recognising the lack of specificity of phenotypic markers alone, we have investigated phenotypic and functional properties for DC using a previously validated ex vivo DC culture system. Using a coordinated and multi-parametric approach, we tested the hypothesis that epithelial cell conditioned media can modulate phenotypic and functional properties of DC.

## METHODS

### Transplant patient clinical data

Clinically stable lung allograft recipients free from chronic allograft dysfunction were used to obtain primary epithelial cells. Clinical details are shown in table 1.

**Table 1:** Clinical details of patients from whom primary epithelial cell cultures were derived

Patient sample	Time after transplant (months)	Diagnosis	Transplant	Age at time of transplant (years)	Biopsy	FEV_1_	BAL microbiology	BOS score
1	12	α_1_-antitrypsin deficiency: emphysema	SL	50.3	A1 B0	1.82	Negative	0
2	6	Primary pulmonary hypertension	HL	52.5	A0 B0	2.57	Negative	0
3	1	Idiopathic pulmonary fibrosis	SL	57.4	A2 B1	1.34	Scanty *E coli*	0
4	12	Idiopathic pulmonary fibrosis	SL	54.4	AX BX	3.87	Scanty *P aeruginosa*	0
5	3	Emphysema	SL	54.9	A1 B0	1.43	Negative	0
6	12	Primary pulmonary hypertension	HL	52.5	A0 B0	2.70	Negative	0
7	6	Primary pulmonary hypertension	HL	60.4	A2 B0	3.09	Negative	0

Biopsy, transbronchial biopsy scored as per international guidelines^12^ for acute and chronic rejection.

BAL, bronchoalveolar lavage; BOS, bronchiolitis obliterans; FEV_1_, forced expiratory volume in 1 s; HL, heart lung; SL, single lung.

### Bronchoscopy and bronchial sampling

Our post-transplant patients undergo surveillance bronchoscopy at 1 week, 1, 3, 6 and 12 months as well as further bronchoscopy if indicated on clinical grounds. All patients underwent pulmonary function testing and an assessment of clinical status was made based on standardised criteria.^10^ Bronchoscopy was performed in accordance with international guidelines.^11^

Bronchoalveolar lavage (BAL) fluid was obtained from either the lingula or right middle lobe and sent for routine microbiological testing. Bronchial brushings (n = 4–6) were obtained from subsegmental bronchi using a standard single-sheathed nylon cytology brush (5 Fr; Wilson Cook, Limerick, Ireland) and dispersed in 5 ml phosphate buffered saline (Sigma, Poole, UK). Transbronchial specimens were taken from either the right or left lower lobe and sent for histopathological examination to exclude acute vascular rejection based on standard ISHLT criteria.^12^

### Primary bronchial epithelial cell culture

Primary bronchial epithelial cells were obtained from bronchial brushings as previously described.^13^ In brief, endobronchial brushings were plated in serum-free bronchial epithelial basal media (BEBM; Cambrex, Wokingham, UK) supplemented with single quouts and antibiotics. When 70–90% confluent, cells were passaged using trypsin to T75 flasks or 24-well plates. When 80–90% confluent, 24-well plates were rested for 24 h in serum-free BEBM media. Epithelial cell conditioned supernatants (ECCM) were then collected following 48 h of culture. Microbiological contamination of ECCM was excluded by testing for normal respiratory flora and fungal and bacterial pathogens in an independent clinically-accredited laboratory.

### Generation of DC and macrophages from monocytes

Monocytes were isolated from freshly drawn peripheral blood from healthy donors or buffy coats, as described previously.^14^ Briefly, peripheral blood mononuclear cells were isolated by density centrifugation on Lymphoprep (Axis-Shield Diagnostics, Dundee, UK). CD14+ monocytes were isolated by positive magnetic selection using anti-CD14 magnetic microbeads (Miltenyi Biotec, Bergisch Gladbach, Germany). Monocytes were cultured at 0.5×10^6^ cells/ml for 6 days in 0.5 ml BEBM and 0.5 ml RPMI-1640 supplemented with 10% fetal bovine serum, 2 mM glutamine, 100 U/ml penicillin and 100 μg/ml streptomycin. To generate DC, IL4 and granulocyte-macrophage colony stimulating factor (GM-CSF) (50 ng/ml each, Immunotools, Friesoythe, Germany) and to generate macrophages (Mph), macrophage colony stimulating factor (M-CSF) (50 ng/ml, R&D Systems, Abingdon, UK) were added to the monocyte cultures on days 0 and 3. After 6 days, cells were stimulated with lipopolysaccharide (LPS, 100 ng/ml, Sigma) or were left untreated. After 24 h the supernatants were harvested and assayed for IL12p70 and IL10 by ELISA, and the DC and MPh were extensively washed before performing functional assays and flow cytometry.

### Effect of ECCM on DC cultures

Monocytes were cultured under DC-generating conditions as described above, but with 0.5 ml ECCM instead of BEBM. The ECCM-modified DC cultures are referred to as antigen-presenting cells (APC) throughout the paper.

### Flow cytometry

The following antibodies were used for cell surface marker analysis: CD1a (NA1/34; Dako, Glostrup, Denmark), CD14 (M5E2), CD68 (27–35), CD86 (2331 FUN-1), CD83 (Hb15e) and HLA-DR (L243; all from BD Pharmingen, San Jose, California, USA). Isotype-matched control antibodies were used to confirm that staining was specific (data not shown). Cells were centrifuged and resuspended in FACS buffer (phosphate buffered saline supplemented with 3% FCS, 2 mM EDTA and 0.01% sodium azide). Human IgG (Grifols, Los Angeles, California, USA) was added with antibodies to prevent Fc receptor binding. To detect intracellular CD68, a permeabilisation step was included (Cytofix/cytoperm; BD Biosciences, Oxford, UK). Cells were incubated on ice for 30 min, washed and resuspended in FACS buffer. Data were collected on a Becton Dickinson FACScan and analysed using FlowJo (Treestar, Ashland, Oregon, USA).

### Phagocytosis assay

Fluorescent-labelled dextran (FITC-dextran; Sigma) was used to assess the mannose receptor (MR)-mediated phagocytic capacity of the cell types. Cells were incubated with FITC-dextran at 37°C for 1 h while a control group was left on ice to exclude extracellular binding of FITC-dextran. Cells were extensively washed and intracellular FITC-dextran was quantified by flow cytometry.

### Mixed lymphocyte reaction (MLR)

1×10^4^ DC, APC or MPh were cultured with 1×10^5^ allogeneic CD3+ T lymphocytes in 200 μl cultures in a 96-well plate. Supernatants were harvested after 6 days and assayed for interferon γ (IFNγ) by ELISA. Proliferation was assessed by incorporation of ^3^H-thymidine for the last 8 h of culture by scintillation counting (Microbeta TriLux, Perkin Elmer, USA).

### Enzyme-linked immunosorbent assay (ELISA)

Cytokine levels in the supernatants were measured by specific sandwich ELISA performed with commercially available matched antibody pairs. The following cytokines were measured: IL8 and IL15 (R&D Systems), IFNγ, IL6, IL10 and IL12p70 (BD Pharmingen).

### Statistical analysis

**Figure 1:**
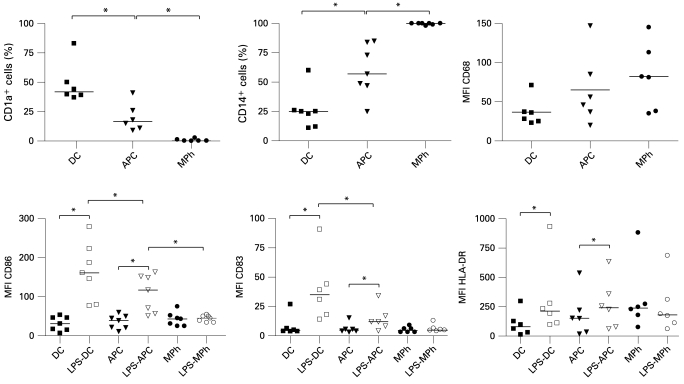
Phenotype of monocyte-derived cell populations. Expression of markers by dendritic cells (DC), antigen-presenting cells (APC) and macrophages (MPh) was assessed by flow cytometry. Lower panels include cell populations activated with lipopolysaccharide (LPS) for 24 h. Debris and dead cells were excluded on the basis of forward scatter and side scatter. Data shown for at least six independent experiments; different epithelial cell donors and blood donors were used in each experiment. Horizontal lines represent median values. *p<0.05 (Wilcoxon test).

**Figure 2:**
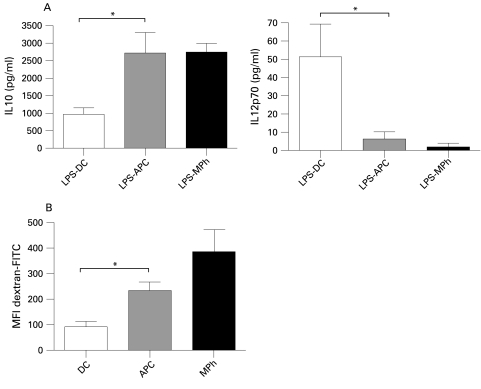
Cytokine production and mannose receptor-mediated phagocytic capacity of monocyte-derived cell populations. (A) Cytokine production. Dendritic cells (DC), antigen-presenting cells (APC) and macrophages (MPh) were activated for 24 h with lipopolysaccharide (LPS, 100 ng/ml). Levels of interleukin (IL)-10 and IL12p70 were determined by specific sandwich ELISA. The mean (SEM) production of six independent experiments is shown. (B) Phagocytic activity. Uptake of FITC-dextran by DC, APC and MPh after 1 h was assessed by flow cytometry. Data are shown as the mean (SEM) of at least three independent experiments. Different epithelial cell donors and blood donors were used in each experiment. *p<0.05 (*t* test).

Statistical software used for the analysis was GraphPad Prism Version 4. In the upper panels of fig 1 the medians of CD1a, CD14 and CD68 expression were compared by the non-parametric paired Wilcoxon test for the following comparator group pairs: DC and APC; APC and MPh. In the lower panels of fig 1 the medians of CD86, CD83 and HLA-DR expression were compared by the non-parametric paired Wilcoxon test for the following comparator group pairs: DC and LPS-DC; APC and LPS-APC; MPh and LPS-MPh; LPS-DC and LPS-APC; LPS-APC and LPS-MPh. In fig 2 the means of IL10 and IL12p70 production and the mean of dextran-FITC uptake were compared by *t* test for the following comparator group pairs: DC and APC; APC and MPh. Only significant results (p<0.05) are indicated in the figures.

## RESULTS

### Bronchial ECCM favour differentiation of monocytes towards a macrophage phenotype at the expense of DC

Table 2 summarises data showing that bronchial ECCM contained a mixture of secreted cytokines including measurable levels of IL8 and IL6. IL15 was not detectable in any of the epithelial cell cultures.

**Table 2:** Summary of IL8 and IL6 measurements made from ECCM

Patient ECCM sample	IL8 (pg/ml)	IL6 (pg/ml)
1	551	31
2	2412	30
3	2809	204
4	340	Not detectable
5	923	84
6	3453	206
7	1472	250

ECCM, epithelial cell conditioned media; IL, interleukin.

Figure 1 shows the expression of markers by the three monocyte-derived cell populations: (1) DC (generated with IL4 and GM-CSF); (2) APC (cultured by adding ECCM to the IL4/GM-CSF DC cultures); and (3) MPh (generated with M-CSF). The effect of a further 24 h stimulation with LPS was assessed for the above cell populations. This is known to activate immature DC, resulting in the upregulation of markers involved in T cell activation.

Phenotypic markers assessed were the DC marker CD1a, the MPh markers CD14 and CD68, the antigen presenting molecule HLA-DR, the classical DC maturation marker CD83 and the T cell co-stimulatory molecule CD86. The salient findings of these experiments showed that ECCM drove the production of MPh-like cells from monocytes. In the APC population there were significantly fewer cells expressing the DC marker CD1a than in the DC population, contrasting with significantly more cells expressing the MPh marker CD14. APC also expressed higher levels of CD68, but this was not statistically significant.

Introduction of LPS stimulation to the protocol led to a significant upregulation of HLA-DR; CD83 and CD86 on both DC and APC, but not MPh. However, the levels of CD83 and CD86 were significantly lower on LPS-activated APC than on LPS-activated DC, while expression levels of HLA-DR were similar.

We also measured the production of the anti- and pro-inflammatory cytokines IL10 and IL12p70, respectively, by DC, APC and MPh that had been activated by LPS for 24 h (fig 2A). APC produced significantly higher levels of IL10 and lower levels of IL12p70 than DC, again resembling MPh rather than DC. Non-stimulated cell populations did not secrete detectable levels of cytokines (data not shown).

### ECCM induce APC that functionally resemble MPh rather than DC

Because the phenotype of APC resembled the phenotype of MPh rather than DC, we hypothesised that the functional characteristics of APC would also be more macrophage-like. To test this hypothesis we assessed functional abilities that are typical for either MPh (phagocytosis) or DC (T cell stimulation).

Figure 2B depicts an assay of mannose receptor (MR)-mediated phagocyte function. Here the MPh represent a positive control as professional phagocytes. Like MPh, ECCM-induced APC exerted greater MR-mediated phagocytic activity than DC.

**Figure 3:**
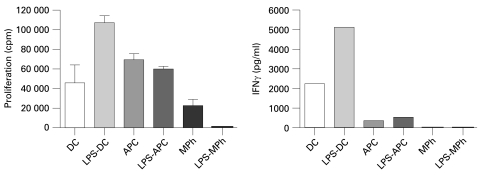
Immunostimulatory capacity of monocyte-derived cell populations. Dendritic cells (DC), antigen-presenting cells (APC) and macrophages (MPh) were or were not activated with lipopolysaccharide (LPS) for 24 h and were used as stimulator cells in a mixed lymphocyte reaction with allogeneic CD3+ T cells. After 6 days, T cell proliferation was determined by ^3^H-thymidine incorporation and interferon γ (IFNγ) production by specific sandwich ELISA. Results are representative of three independent experiments and are shown as mean (SEM). Different epithelial cell donors and blood donors were used in each experiment.

Figure 3 summarises the results of mixed lymphocyte reactions to assess the immunostimulatory capacity of the monocyte-derived populations. Here LPS-activated DC represent a positive control as professional APC, potently initiating T cell responses. Activation of DC with LPS resulted in enhancement of T cell stimulatory activity of DC, with higher induction of proliferation and production of the prototypic cytokine IFNγ by allogeneic T cells. This enhanced T cell stimulatory capacity of LPS-DC is consistent with the higher expression of CD83 and CD86 and the production of the Th1-inducing cytokine IL12p70. In contrast, activation of APC with LPS did not enhance their immunostimulatory capacity, and the induction of IFNγ production was greatly reduced compared with DC and LPS-DC. MPh were the poorest stimulators of T cell proliferation and only induced very low levels of IFNγ. These experiments show that ECCM-induced APC have a low immunostimulatory capacity compared with DC.

Together these data indicate that ECCM skew monocyte-derived DC towards a macrophage-like population in terms of phenotype and function.

## DISCUSSION

This study has shown that conditioned media from lung allograft primary bronchial epithelial cells drives the production of macrophage-like cells from monocytes rather than DC. Although there was biological variability, a broad range of phenotypic and functional markers consistently supported this finding. Our data suggest that epithelial cells from allograft recipients promote production of MPh while restraining the airway DC population and potential alloimmunity. It is unclear whether the observed effect is specifically seen in lung transplant recipients or might be a general property of bronchial epithelial cells. It remains to be studied whether this is relevant both to lung allografts and to the lung more generally.

The biology of professional APCs in the airway is likely to be of key importance in the alloimmune component of chronic allograft dysfunction which is commonly referred to as chronic rejection. There are few studies in lung transplantation, however, and these are restricted to phenotypic approaches using different markers which report conflicting data. Two studies indicate that, in chronic rejection, there are increased numbers of DC^3^ ^4^ with an earlier study from our group indicating lower numbers of CD1a+ DC in lung allografts.^5^

It is accepted that the local environment to which DC are exposed is a key factor in their maturation and function.^8^ The close spatial relationship between airway epithelial cells and DC indicate that epithelial cells might be an important influence.^8^ Airway epithelial cells secrete a spectrum of proinflammatory cytokines and growth factors which play an important role in local defence and in the induction of a systemic inflammatory response. In lung allograft primary cultures we have previously reported that airway epithelial cells constitutively secrete GM-CSF, M-CSF and IL6,^6^ factors that increase the production and mobilisation of monocytes from the bone marrow and influx into the lung. In ex vivo models where DC are produced from monocytes GM-CSF is used in the conditioning protocols, emphasising the potential for epithelial cell-derived products to influence DC differentiation. Further research is required to define the epithelial cell factor(s) interfering with DC differentiation. We considered the possibility that the anti-inflammatory cytokine IL10 was involved, but could not detect this cytokine in ECCM by ELISA (lower limit of detection 15 pg/ml, data not shown). Another possibility is IL6, which was detectable in the majority of the ECCM used in this study. It has been reported that high levels (>50 ng/ml) but not low levels of IL6 (20 pg/ml) switch monocyte differentiation from dendritic cells to macrophages.^15^ Because ECCM contain only moderate levels of IL6 (30–250 pg/ml, table 2), we feel it is unlikely that IL6 by itself was responsible for the observed inhibitory effects on DC differentiation, although it cannot be excluded that IL6 synergised with other soluble ECCM component(s).

The potential for epithelial cells to interact with DC was recently studied by Regamey *et al* using lung A549 and airway BEAS-2B epithelial cell lines.^9^ Conditioned media from the epithelial cells contained IL15 both constitutively and following stimulation with inflammatory stimuli. Addition of these media to monocytes led to an upregulation of IL15 receptor mRNA expression, with differentiation of monocytes to functional DC.^9^ In the allograft setting, where upregulated alloimmunity is damaging,^1^ ^16^ these findings are of potential significance, but there are no previous data of which we are aware in primary airway epithelial cells from allograft patients.

Our findings are not consistent with those of Regamey *et al*. Using accepted markers for DC and macrophages we consistently showed that conditioned media from allograft recipient primary epithelial cells favoured the production of macrophage-like cells from monocytes. Phenotypic markers were corroborated by functional phagocytic and immunostimulatory assays. Our data showed that incubation of monocytes with patient-derived ECCM consistently yielded a population of cells with low expression of DC markers and poor T cell stimulatory ability. In contrast, we observed significant expression of macrophage markers and competent phagocyte function. The conditioned media from allograft primary epithelial cells did not contain detectable levels of IL15 as measured by ELISA (data not shown), which may reflect differences between primary epithelial cells and cell lines and/or differences in the experimental set-up (eg, cell density).

A main difference between our work and the study by Regamey *et al* was that we used primary epithelial cells sourced from lung transplant recipients. It is of interest that a previous study by Ohtoshi *et al* using upper airway epithelial cells from polyps drove the production of MPh from monocytes, although this was in atopic but otherwise healthy volunteers and not a transplant-related study investigating DC markers.^17^ More recent work involving an airway epithelial cell line and murine DC has also indicated that epithelial cells may modify immune responses by inducing an “anti-inflammatory” DC-suppressing microenvironment.^18^ This may indicate that our data which as far as we are aware, are the first in primary human cells, have relevance beyond lung transplantation. Promoting the production of MPh-like cells is arguably physiologically appropriate in the human airway since MPh form an important defence in the constantly exposed lungs. The antimicrobial activity of MPh is promoted by efficient phagocytosis and, together with the barrier function provided by the tight airway epithelium, MPh represent a first line of innate immunity.^19^

Danger signals provided by stimuli such as infection in the allograft may be important in promoting alloimmunity.^16^ The emerging paradigm is that non-alloimmune damage due to potentially occult challenges such as infection and aspiration may combine with alloimmunity to yield damage.^16^ The airway epithelium is a target for injury, with damage and loss of barrier function in chronic allograft dysfunction. Our recent finding of decreased levels of the epithelial cell product secretory leukocyte proteinase inhibitor (SLPI) in the BAL fluid of lung allograft recipients with bronchiolitis obliterans supports this model.^20^ In addition, our study showed that the antimicrobial peptide LL-37 is elevated in the BAL fluid of allograft recipients with chronic dysfunction.^20^ As well as being an antimicrobial, LL-37 has been shown to interact with DC, and CD86 expression following LL-37 treatment has been shown to be increased in a dose-dependent manner.^21^

In the present study we showed that monocytes incubated with ECCM and then stimulated with the Toll like receptor 4 agonist LPS upregulated HLA-DR, CD83 and CD86, which are markers of DC activation. However, the T cell stimulatory capacity of ECCM-induced APC was not enhanced. It is likely that the high production of the anti-inflammatory cytokine IL10 by LPS-activated APC will have counteracted the T cell co-stimulatory activity of CD83 and CD86, but this requires further investigation. Interestingly, the ECCM-induced APC resemble regulatory DC (eg, high IL10 production, low T cell stimulatory capacity) described previously by us and others,^14^ ^22^ ^23^ but whether they are capable of inducing T cell tolerance requires further investigation.

Our data emphasise the need for primary cell data to be assessed along with experiments involving cell lines, although primary studies are themselves limited by technical difficulty and the fact that they are resource intensive. Further studies would be useful to investigate conditioned media sourced from epithelial cells from subjects with chronic allograft dysfunction. This was beyond the scope of this present study, since our experience is that such cultures commonly fail due to antibiotic-resistant patient-derived infection.^13^ Such work may require specialised protocols and augmented—possibly patient-specific—antibiotic approaches.

Our experiments were all performed using epithelial cells at early passage (I–II) in cell cultures free from transplant maintenance therapies. We do not expect previous patient immunosuppression to be responsible for our findings, but transplant medications might affect the interplay between epithelial cells and DC in the airway. This could also be an avenue for further research. An aim of this could be to elucidate the most appropriate balance between appropriate immunosuppression and preservation of airway homeostasis, with maintenance of epithelial function and appropriate networking with airway DC representing a desirable goal.
